# Evaluating Changes in Health Risk from Drought over the Contiguous United States

**DOI:** 10.3390/ijerph19084628

**Published:** 2022-04-12

**Authors:** Babak Jalalzadeh Fard, Jagadeesh Puvvula, Jesse E. Bell

**Affiliations:** 1 Department of Environmental, Agricultural, and Occupational Health, College of Public Health, University of Nebraska Medical Center, Omaha, NE 68198, USA; jagadeesh.puvvula@unmc.edu (J.P.); jesse.bell@unmc.edu (J.E.B.); 2 Daugherty Water for Food Global Institute, University of Nebraska, Lincoln, NE 68588, USA; 3 School of Natural Resources, University of Nebraska-Lincoln, Lincoln, NE 68588, USA

**Keywords:** drought and health, drought risk, Local Moran’s I, drought vulnerability, U.S. drought

## Abstract

The outcomes of drought can be difficult to assess due to the complexity of its effects. While most risk assessments of drought are developed for agriculture or water resources, the associations with human health are not well studied due to unclear and complex pathways. This study is the first to assess potential changes in health risk from droughts during the last decade in the contiguous United States. To assess the risk, we spatially superimposed vulnerability variables associated with drought on historical drought exposure over the last decade. Different variations in Local Moran’s I statistics were used to assess the spatial distribution of health vulnerability, risk of drought, and changes in the two five-year study periods (2010–2014 and 2015–2019). Our results show large clusters of the western United States had a significant increase in risk during the latter part of the study period due to increases in vulnerability and hazard. In addition, southern areas of the United States were consistently above the national average in drought risk. Since our vulnerability variables include agriculture, drinking water, and sociodemographic indicators, the results of this study can help various experts interested in drought preparedness efforts associated with human health.

## 1. Introduction

Drought is characterized by a lack of precipitation for a prolonged period during the natural climate cycle [1]. During the past several decades, extended dry periods have become more frequent in parts of the United States (USA) (such as the Southwest) and these patterns are predicted to continue in the future [2,3,4,5]. Over forty-one years (1980–2021), there were 29 drought events recorded in the U.S. that caused severe economic impact (totaling USD 285.4 billion) and loss of life (totaling 4139 deaths) [6]. Multiple studies have connected drought to a variety of negative human health outcomes [7,8,9,10]. The human health outcomes of drought extend to both physical and behavioral health [11]. As droughts are expected to increase over time due to human-caused climate change, it is important to understand the current risks to improve future preparedness efforts [12,13].

The Intergovernmental Panel on Climate Change (IPCC) Special Report on Managing the Risk of Extreme Events to Advance Climate Change Adaptation (SREX) was designed to mix the fields of climate adaptation and risk management [14,15]. SREX defines the risk of natural hazards as the dynamic interaction between climate-related hazards (such as heatwave, flood, drought, etc.) with the exposure and vulnerability of affected humans or the ecological system to the hazard. From this definition, the health risk of a climatic event can be considered as the interaction of the considered natural hazard with the vulnerability variables that can mediate the hazard into potential health effects depending on the exposure of the vulnerable groups.

The human health risks associated with droughts are typically due to interactions between intensity/duration/frequency of drought (hazard), local impact of drought (exposure), susceptibility (vulnerability), and capacity to cope (adaptive capacity) [16,17]. Population vulnerability is especially important, as certain community characteristics can make populations more susceptible to adverse impact from environmental hazards (such as drought) [10]. Drought vulnerability has generally been linked to poverty [18]. Specific drought-related health outcomes associated with air quality, airborne illnesses (such as Valley fever), and food insecurity [19,20,21,22,23,24] have also been associated with poverty. Reliance on small or poorly maintained water systems puts populations at increased risk of morbidity due to exposure to contaminated drinking water or issues resulting from reduced use of water resources for hygiene and food washing [3,21]. Children and the elderly are both vulnerable to various drought-related health outcomes, such as air- and waterborne diseases [22,24,25]. Seniors living in care facilities can also experience morbidity due to water-related stresses on electricity and HVAC systems [3,25].

Studies have also demonstrated that youths are vulnerable to adverse mental health effects in rural areas [24]. Populations reliant on agriculture for livelihoods or sustenance are vulnerable to food insecurity, malnutrition, and the accompanying adverse mental health effects when drought causes production to suffer [3,9,18,24]. In addition, lowered surface water volumes put individuals using recreational water at risk of waterborne disease and injury from swimming or boating accidents [3]. The drought-related hazards and exposures are often expressed using the spatiotemporal information of the drought event. Vulnerability factors related to poverty and income, technology, education, and infrastructure were found to be relevant to drought [26,27,28]. For example, the interaction between water scarcity (infrastructure vulnerability) and drought events could have a serious impact on mental health among farmers, mediated by impact on agriculture [1,11,29]. Vins et al. (2015) reported on the causal relationships between drought and a variety of mental health outcomes [9].

**Figure 1:**
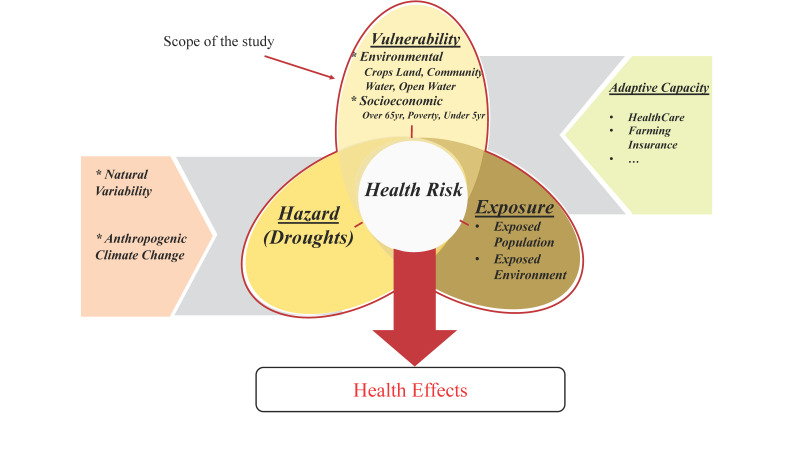
Adaptation of SREX framework for health risk of drought. The red boundary shows the scope of this study.

The above-mentioned studies all consider measuring the direct effects of drought on different health outcomes. While these studies are necessary, a gap exists in assessing the distribution of community measures of vulnerability and their exposure to drought. Given this predicted increase, our study adapted the SREX risk framework (Figure 1) and used a snapshot of data to identify locations that are vulnerable to negative health effects of drought and their temporal changes in vulnerability variables and health risks. This can help target current public health interventions to mitigate these effects in areas that are more susceptible. Through this study, we wish to determine how health risks of drought can change in the contiguous United States and what locations are under higher levels of risk.

## 2. Materials and Methods

We examined 10 years (from 2010 through 2019) of drought over 3108 counties in the contiguous United States (CONUS). This timespan covers two non-overlapping American Community Survey (ACS) 5-year estimates and therefore provides an opportunity to evaluate changes between the two periods [30]. The ACS is conducted by the U.S. Census Bureau to understand changes in social, economic, demographic, and housing characteristics of the United States. We chose ACS 5-year estimates due to the highest accuracy and inclusivity among ACS datasets, while other products are restricted to a population of 20,000+ (for 3-year and 1-year supplemental estimates) or 65,000+ (for 1-year estimates). This includes 3108 (all) counties over 48 CONUS states and the District of Columbia. We then chose other datasets from times that best fit into these two periods.

We created our hazard parameters from the U.S. Drought Monitor (USDM) weekly drought categories of extreme and exceptional drought episodes (D3 and D4) and evaluated human health risks associated with drought exposure using different variations in Local Moran statistics (generally known as Local Indicator of Spatial Association (LISA) [31]).

USDM is a composite index built on 40–50 input parameters that include drought indices, soil moisture, hydrological, climatological, and modeled/remote-sensed metrics. Svoboda et al. (2002) emphasized the usefulness of USDM across agricultural production, availability of water resources, and wildfires [32,33]. Since USDM is the current standard for drought monitoring and is used for broad applications, we considered this index to be ideal in our multidisciplinary study exploring the association between drought and health risks.

### 2.1. Data

#### 2.1.1. Vulnerability Variables

Environmental and socioeconomic factors that cause certain populations to be predisposed to adverse effects of drought are known as vulnerability variables. In our adapted framework, vulnerabilities are characteristics of the system that make it experience more severe effects when exposed to hazards. Adaptive capacities are different coping mechanisms that can reduce the effect of hazards, such as medical care facilities or water management plans for droughts. Looking into related literature (as mentioned in the Introduction), we considered six vulnerability variables to create the basis for an estimation of drought vulnerability for this study. From the adapted framework, the vulnerability variables are characteristics of the exposed system (the related population and environmental characteristics of the counties) to the hazard (drought) that cause more adverse outcomes (number of morbidities or mortalities). In this framework, health records of attributable diseases can be later used to evaluate the output of this study or to measure the success of adaptation strategies (Health Effects in Figure 1). Vulnerability variables for this study involved three demographic measures (the ratio of populations over 65 years of age, under 5 years of age, and below the poverty level), two environmental measures (area proportions of cropland and open water), and a determinant of the population in a higher risk of being affected by drinking water contamination (Water Quality) during drought periods.

We used tidycensus package in R statistical software to download the ACS 5-year total population and population over 65 years old, under 5 years old, and below poverty line for each county in CONUS to calculate ratios in each study period [34,35]. To estimate the effect of water quality, we used data from the Environmental Protection Agency’s Safe Drinking Water Information System (SDWIS) [36]. We downloaded total populations and populations below 3300 people (representing small communities) from Pop Cat 3 categories in the SDWIS system for 2014 and 2019 (representing each study period) and calculated the rate of small community populations for each county in each study period.

Populations that rely on agriculture for livelihood or sustenance are vulnerable to food insecurity, financial impact, and the accompanying adverse mental health effects when drought causes production to suffer [3,9,24,37]. Lastly, lowered surface water volumes put recreational water users at risk of waterborne disease and injury from swimming or boating accidents [3]. To incorporate these two effects, we downloaded the National Land Cover Database (NLCD) for 2016 and 2019 for the first and second study periods, respectively [38]. We then used Zonal Statistics in ArcGIS Pro to aggregate the 30 × 30 m cells to county-level and calculate ratios land type by cultivated and open water in each county from each of the NLCD products [39].

The final step included using the unique Federal Information Processing System (FIPS) Codes for each county to aggregate the six vulnerability variables into two datasets corresponding to each study period.

#### 2.1.2. Hazard Parameters

Natural hazards can affect systems through different mechanisms. These mechanisms are usually measured by three parameters of intensity, duration, and frequency (IDF). As expected, more intense hazards cause more severe effects. Longer periods of hazard can accumulate the effects on the system and create a higher impact in the end. Lastly, more frequent hazards may create new stress on a system that is not fully recovered from the previous hazard, causing higher damage than a restored condition. A well-known example of using these hazard parameters is the use of intensity–duration–frequency (IDF) curves in hydrology studies to estimate flood hazards [40]. Considering the three explained mechanisms of a hazard, we used the USDM weekly index over the study period to create continuous hazard parameters that represent the intensity–duration–frequency of drought in each study period.

To account for the intensity of the drought, we only considered weeks in D3 (extreme drought) or D4 (exceptional drought) categories as intense hazard periods. The duration parameter for each 5-year study period was calculated as the number of weeks in intense hazard (USDM D3 or D4) divided by the total weeks in the study period. The frequency parameter for each study period was calculated as the ratio of maximum consecutive weeks of intense hazard (USDM D3 or D4) to all weeks of the study period. This gave us two continuous hazard parameters in each county for each study period, and the total hazard was calculated by adding these two parameters.

In calculating the changes in each hazard parameter over the study periods, we performed a two proportions z-test with 0.05 significance level for each case and replaced the hazard changes with non-significance z-tests by zero [41]. Using this test, we compared whether the hazard level had changed for each county over the study periods. For each county, there were two sets of weekly drought levels for each study period, and our hazard parameters declared the number of weeks in the intense hazard condition. Therefore, each hazard parameter in the first study period by county was compared with its counterpart for the second study period using this test with a null hypothesis that the proportions are the same (0.05 significance level). Non-significant values indicate that the hazard parameter did not changed between the two study periods.

### 2.2. Methods

#### 2.2.1. State-Level Changes in Vulnerability Measures

We used Brown–Mood median test to compare state-level changes in each vulnerability variable over the study periods [42]. The Brown–Mood test compares the values in each group with the global median of the accumulated group values. In our case, each test compared the median county value of each vulnerability measure for a study period to the corresponding median county value in the other study period. The null hypothesis is that the medians of the populations from which the groups sampled are equal; therefore, the significance is when the medians of the two groups are different. The sample sizes varied from a minimum of 5 counties in Rhode Island to a maximum of 254 in Texas. These provided a general overview of state-level changes in vulnerability variables. Coin package in R was used for conducting the Brown–Mood test [43]. We then summarized the results by removing non-significant cases and calculating the changes in medians for each vulnerability variable over each state for significant values (vulnerability median in 2015~2019—vulnerability median in 2010~2014). The results are changes in vulnerability variables (as represented by ratios) over the study period. A threshold of α = 0.95 was used for the significance of all statistical tests in this study.

#### 2.2.2. Local Moran’s I Statistics

We used different variations of Local Moran statistics to assess the spatial association of different study variables (vulnerability variables or hazard parameters) in each county with the average values of the same or other variable in its neighboring counties [31]. Local Moran statistics is one of the known methods of Local Indicator of Spatial Association (LISA). The values of I and the significance levels are calculated as below:

(1) $$I_{i}=A\cdot z_{i}\;\sum_{j}w_{ij}z_{j},$$

(2) $$\left(pseudo\right)\;p_{i}=\frac{n_{i}+1}{m+1}$$

where *z* are normalized values of the considered variable centered on its mean; *i* represents the county for which *I* and *p* are calculated; *I_i_* and *p_i_* are observed Moran statistics and the significance level at county *i*, respectively; and *Wij* are average weights from contiguity matrix that distinguish the neighboring counties to county *i*. For this study we used the Queen contiguity algorithm to create the contiguity matrix [44]. Queen contiguity considers all counties that have common borders or common edges with each county as its neighbors. *A* is a constant value in each set of LISA calculation, computed as $A=1/\sum_{i}z_{i}^{2}$. The summation part in Equation (1) is known as spatial lag and measures the effect of neighboring counties through the weighted sum of their considered variable. Equation (2) calculates the pseudo *p*-value for each county, where *n_i_* is the number of times that calculated *I* from permutation is higher (or lower when *I_i_* is negative) than *I_i_*, and *m* is the total number of permutations. For more details on Local Moran’s I calculations, we refer the reader to Anselin (1995) or documentation in GeoDa software. We used GeoDa software package version 1.18.0.16 on Mac operating system for all LISA analyses in this study [31,45]. Usually, spatial analyses methods are prone to the problem of multiple comparisons due to spatial dependencies [46]. This problem causes a large number of false positives—where a true null hypothesis is incorrectly rejected—because of the spatial dependence of nearby data. We addressed this problem by testing the three available remedies in GeoDa (Bonferroni, false discovery rate, and adjusting the significance threshold) and chose the one that resulted in the least conservative results. Therefore, each analysis considered pseudo *p*-values of 0.01 or lower as significant for remedying the multiple comparison effect and a significance level of 0.05. Each LISA analysis was conducted with *N* = 9999 permutations. The results of Moran’s I analysis were categorized into four significant groups and one non-significant group. The significant categories are named by two-word combinations of ‘High’ or ‘Low’ for (variable|spatial lag). ‘High’ and ‘Low’, respectively, mean higher or lower than the average of the corresponding variable (or spatial lag) among all samples in the test (counties for our study). (High|High) or (Low|Low) combinations show clusters—similar relations with means (higher or lower) for both variable and spatial lag—while (High|Low) and (Low|High) represent outliers—opposite relations with means for the variable and spatial lag-.

To incorporate the vulnerability variables into the analysis, we transformed each vulnerability value into its percentile rank by overall counties following the method used by the CDC for creating their Social Vulnerability Index [47]. Total vulnerabilities in each study period were then calculated by adding ranked vulnerabilities in each county and a final percentile ranking over the summed values.

We captured the spatial clusters and outliers of changes in vulnerability variables over the two study periods using the Moran’s I analysis of vulnerability changes. These are also known as Differential Moran statistics. We then mapped the spatial distribution of risk for each pair of hazard parameters and vulnerability variable in each study period using bivariate Local Moran’s I analysis with vulnerability as the variable and the hazard parameter as spatial lag. We calculated the adjusted risk in each state by first calculating the ratios of counties resulting as high (vulnerability) and high (hazard) (high risk) to the total counties of that state and then dividing that ratio by the similar national level ratio. This process was applied to each combination of vulnerability and total hazard in each study period, and the results produced adjusted risk maps. In the next step, we identified areas with significant total risk in each study period using bivariate Local Moran’s I with total vulnerability as a variable and the normalized total hazard—addition of the two hazard parameters—as the spatial lag. In the last step, we mapped the changes in total risk over the study periods. For this purpose, we used differences in total vulnerability values between the study periods as the central variable and the normalized values of changes in total hazard values for spatial lag. Figure S1 in Supplementary Materials provides flowcharts of the different methods described in this section.

## 3. Results

### 3.1. Changes in Hazard

**Figure 2:**
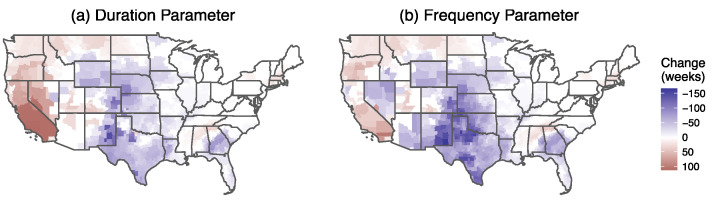
Changes in hazard parameters from the first study period (2010–2014) to the second study period (2015–2019). Negative values show a significant decrease from the first to the second study period. Positive values indicate a significant increase in the second study period.

Geographic variability was plotted for the United States (Figure 2). Increased hazard levels were observed in the West and Northwest. Southern California experienced the highest increases in both parameters measured. Of the 32 counties in the 99th percentile of duration parameter, 27 are located in California and 5 in Nevada. The highest increase in the frequency parameter occurred in 19 California counties and 8 Arizona counties. The state with the largest increase was California—a 100-week increase. Decreased levels in the Central U.S. were distinguishable for both duration and frequency parameters. Maps of each hazard parameter for each study period are provided in the Supplementary Materials (Figure S4).

### 3.2. Vulnerability Variables

State-level comparisons of vulnerability variables showed significant changes in 16% of total combinations (46 out of 6 vulnerabilities for 48 states) in the two study periods (Table 1). It consisted of three of the six studied vulnerability measures. The population gets significantly older in 26 Eastern states and experiences a significant reduction in the percentage of those under 5 years of age in 7 states. There was also a decrease in the percentage of the population below the poverty level in 13 states. Looking into the spatial distribution of these changes, the decrease in those under 5 years of age occurred in the southeast region and Utah (Figure S2 in Supplementary). An increase in the population of those over 65 years of age occurred in the East and Utah. States with the highest decreases in the percentage of the population below the poverty line were in the eastern part (Figure S2).

**Figure 3:**
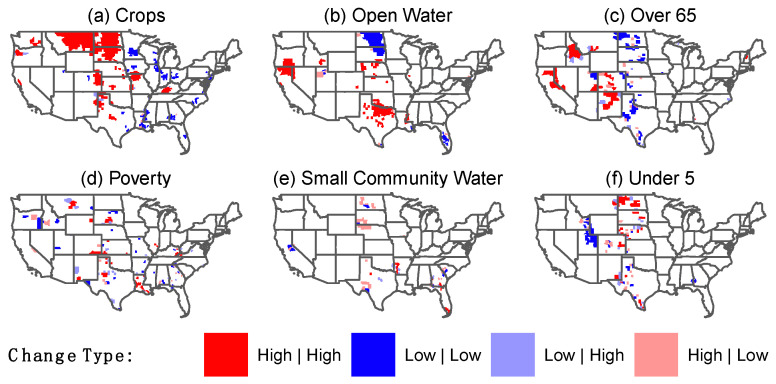
Results of Local Moran’s I analysis for the changes in vulnerability variables over the study periods. The six figures show changes in vulnerability over the two study periods (2010–2014 to 2015–2019). Dark red indicates the clustered increase and dark blue indicates a clustered decrease. Light red and light blue indicate high outliers and low outliers, respectively.

Except for two of the vulnerabilities (percentage of crop-land and percentage of open water), the spatial distribution of vulnerability change between study periods did not capture large clusters (High|High or Low|Low) (Figure 3). Figure 3 depicts the results of Differential Local Moran’s I analysis on vulnerability changes over the study periods. Three large clusters of increase in crop-land ratios are recognizable in the Northern Rockies and Plains climate region (from the nine U.S. climate regions identified by the National Oceanic and Atmospheric Administration (NOAA)) that contain 40.9% (27/66), 75.4% (40/53), and 58.9% (33/56) of counties in states of South Dakota, North Dakota, and Montana, respectively (Figure 3a) [48]. These increases represent the change in area of cultivated crops lands that cause higher vulnerabilities. Therefore, it cannot be attributed to the effect of drought. North Dakota experienced the largest decrease in open water, with 62.3% (33/53) of its counties in the Low|Low cluster group that extended into northeast South Dakota (with 21.2% (14/66) counties involved) (Figure 3b). The increase in hazard parameters for North Dakota (Figure 2) is too small to be the driver of such changes in open water. These changes can be the result of different water management strategies or other unidentified reasons. Other vulnerability measures in Figure 3 do not contain clusters as large as those found in the two previously mentioned. Supplementary Figure S3 provides raw values for vulnerability changes categorized in the box-plot mapping schema. Comparing this result with the results from Table 1 shows the importance of carrying out spatial analyses in the local scales for revealing such spatial patterns.

### 3.3. Health Risks

**Figure 4:**
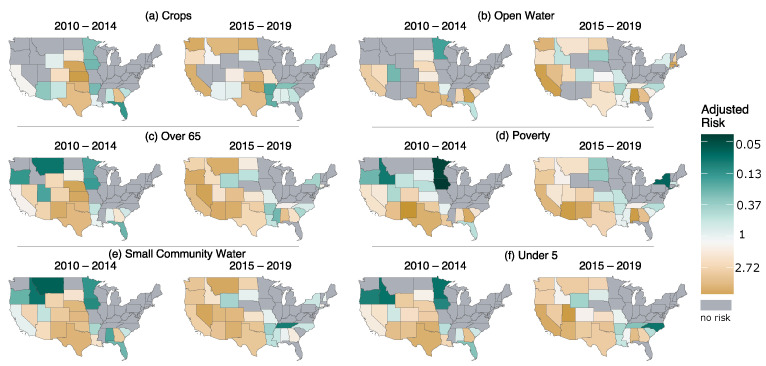
Adjusted risk values for each state. Gray states contain no counties in (high vulnerability|high hazard) risk condition. Darker green values indicate that the state has a lower risk, and brown indicates a higher risk. Values are calculated by dividing the state level ratios of a number of counties in high vulnerability|high hazard clustering to all counties to the similar national level.

Parts of the southern United States show above-average adjusted risk levels for most vulnerabilities during both study periods (Figure 4). Grey states in Figure 4 correspond to cases where no counties registered as having high vulnerability and high hazard clustering. Texas persistently shows adjusted risk over 1 for all the cases, while its hazard levels for the second period are not as high as the West and Southwest (Figure S4). The increase in vulnerability patterns for crops and open water in the Northern Rockies and Plains (captured in Figure 3) affected the corresponding risk increases in the second period (Figure 3a,b). East and Northeast show no risk or risk below the national average in all cases. California, Oregon, and Washington experienced higher than average risks for the second study period and increases from the first period. The Central region (Nebraska, Iowa, South Dakota, Kansas, and Missouri) experienced decreases in risk ratios in the second period for all vulnerabilities, indicating that no counties were categorized as high vulnerability and high hazard. From the top agriculture producing states (California, Iowa, Nebraska, Texas, Kansas, Minnesota, Illinois, Wisconsin, Indiana, and North Carolina) in 2020, Texas persistently experienced higher than national average risk in all combinations of vulnerability in both study periods. In a broader view, the effect of the shift of hazard into the west for the second study period (Figure 2) affected the final risk levels in all six vulnerability measures in Figure 4.

**Figure 5:**
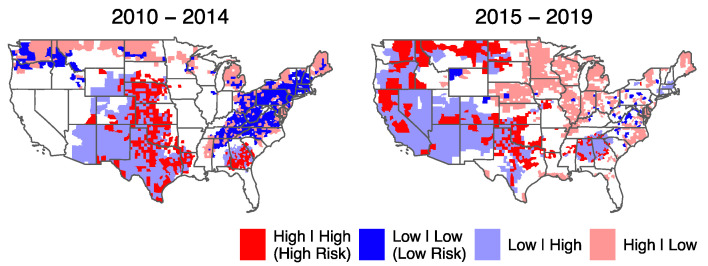
Total risks in each study period. Results of bivariate Local Moran’s I analysis with total vulnerability as the variable and total hazard as spatial lag. High (Low) indicates higher (lower) than the total average of the corresponding variable over all counties.

Bivariate LISA analysis of total risks indicates a considerable spatial shift of high-risk areas from the South to the Midwest (33% (219/657) of counties in the South and 20% (60/291) in the Northern Rockies and Plains) in the first period to the Southwest (28% (21/75) of counties in the West, 28% (33/119) counties in the Northwest, and 13% (37/291) in the upper Northern Rockies and Plains) in the second period (Figure 5). Risks were measured by the four categories of vulnerability and hazard combinations, with total vulnerability as the central variable and the total hazard of surrounding counties as spatial lag. The figure shows concentration of high risk and low vulnerability with high hazard counties in the south and central areas of the United States during the first study period. In the second period, the Western states of California, Oregon, and Nevada also experienced high hazard. The north section of the United States experienced high shifts from low hazard areas in the first study period into high hazard areas in the second. These figures can give a general understanding of the spatial distribution of total risk in each study period, while specific areas within each state can be focused on for further studies.

**Figure 6:**
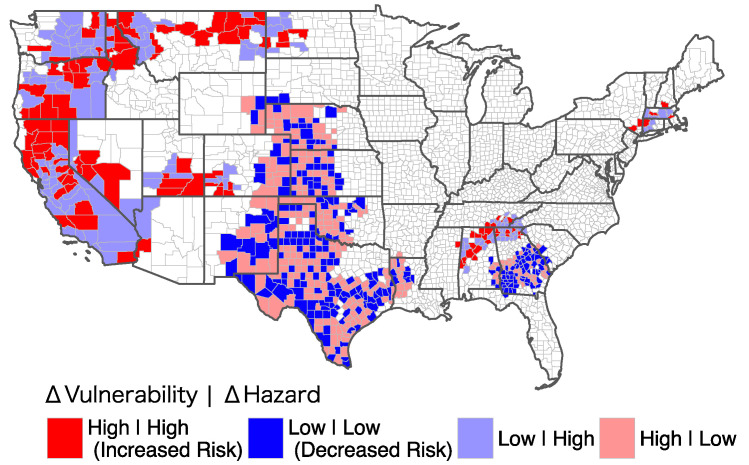
Changes in total risk of drought from the first to the second study period. The maps show the results of bivariate differential Local Moran’s I analysis with change in total vulnerability as the main and change in total hazard as spatial lag variables. High (Low) indicates higher (lower) than the total average of the corresponding variable in all counties.

Estimating changes in total risk distinguished two large regions with opposite patterns of change (Figure 6). All western shoreline states, along with northern Idaho and roughly half of Montana, contain a large region of low or high change in vulnerability—meaning the change in vulnerability is lower or higher than average, respectively. This change is influenced by a change in levels of high hazard. A total of 53% (31/58) of counites in California show low to high change in drought risk, and 40% (23/58) experienced increased risk. A total of 93% of counties in California experienced above-average change in total hazard levels, distinguished by ‘High’ as the second part of the legend in Figure 6. There were 33.3% (12/36) of counties in Oregon that experienced increased risk, and 36.1% (13/36) placed in Low|High category. The state of Washington showed 12.8% (5/39) and 43.6% (17/39) in the increased risk and ‘Low|High’ categories, respectively. From the southern borderline of Texas to northern Nebraska contained randomly distributed counties with low or high total vulnerability change levels inside a low hazard change region that covers most of the South, part of the Northern Rockies and Plains, and Southwest climate regions.

**Figure 7:**
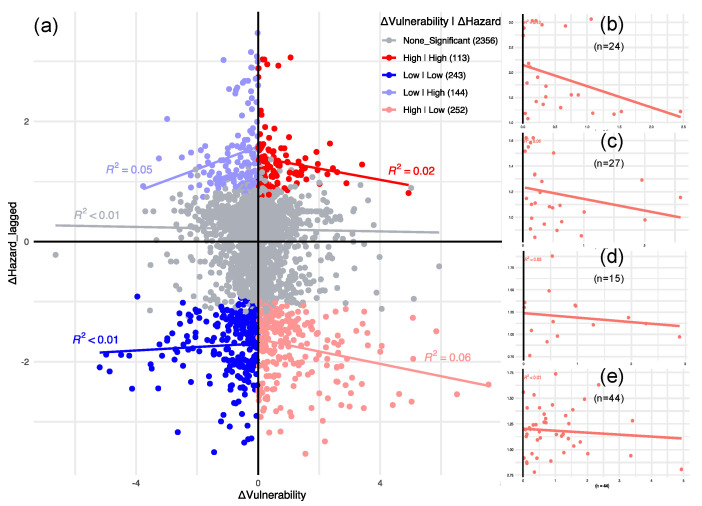
Moran’s I scatterplot of changes in total risk. (**a**) All counties, (**b**–**d**) regional subgroups of counties in the High|High (increased risk) category with (**b**) California, (**c**) Southeast, (**d**) Central, and (**e**) Northwest.

For an increase in risk, the rate of increase in hazard to increase in vulnerability showed a decline, since the higher increase in hazards generally corresponds to a lower increase in vulnerability (upper right quarter of Figure 7a). Figure 7 depicts the Moran scatterplot of the analysis presented in Figure 6, with each point representing a county, the x-axis capturing standardized change in total vulnerability, and the y-axis showing the spatial lag of standardized changes in total hazard. We further investigated subgroups of increased risk change and their trends (Figure 7b–e). California (Figure 7b) showed the most promising trend among other states with the sharpest decline in the regression line. The Southeast region has a relatively sharper decline (Figure 7c) compared with Nevada, Utah, and Colorado (Figure 7d) and the areas of covering North Dakota, Minnesota, Idaho, Washington, and Oregon (Figure 7e).

## 4. Discussion

Our study explored how vulnerability variables and human health risks associated with drought changed over CONUS from 2010 to 2019. The results of this study indicate that large areas experienced significant changes in risk. Large portion of the western United States had increases in drought hazard levels in areas with both decreased and increased changes in drought vulnerabilities. A better understanding of changes in drought vulnerability in the western United States is important due to the projected increase in drought frequency for that region due to anthropogenic climate change and the identified health outcomes associated with drought in this region [49,50]. Between the two study periods, hazard levels decreased in Texas, New Mexico, Oklahoma, Kansas, and Nebraska. However, vulnerabilities increased and decreased in the South and Midwest (Figure 7). Our change in vulnerability maps (Figure 3) reveal areas of higher concern for drought impact. Further investigations into the underlying mechanisms of such changes require regional focus and studies and specialization in related disciplines. In addition, comparisons of vulnerability variables reveal that half of the states had significant increases in elderly populations that are potentially more vulnerable to drought [51]. Using the Moran scatterplot, we were able to determine the trends between change in hazard change and change in vulnerability in each category of risk. These results, especially for the increased risk group and its subgroups, can assist with regional planning efforts, where several counties have different grouping and may need prioritization based on trends besides their categories of change in risk.

Our results show that multiple factors must be considered when evaluating the risk and vulnerability of climate. The 2021 Special Report on Drought by the United Nations Office of Disaster Risk Reduction (UNDRR) considers such mapping key for developing systemic risk governance mechanisms [29]. Both indicators of vulnerability and distribution of the hazard changed between two periods in a single decade. Reevaluation of both vulnerability and risk should be carried out on a regular basis to understand potential changes in threats. Risk mapping can help develop scenarios for identifying priorities at global, national, regional, and local scales [16,52,53,54]. Total risk maps can help prioritize areas for adaptation or resilience-building efforts, while individual risk maps can identify which vulnerability measures are more exposed [29]. Hazard mapping can direct the need for potential mitigation strategies, such as water management plans or transboundary water treaties [29]. An increase in vulnerabilities can help determine where to strengthen adaptation capacities, such as considering public health measures to reduce threats of forecasted droughts. For example, an increase in cultivated crop-land in Montana, North Dakota, and South Dakota (Figure 3a) indicates an increase in vulnerability. Looking into the change in hazard (Figure 1) in these states also shows an increase. Since risk in our framework (Figure 1) is the exposure of vulnerability to hazard, we can expect that the risk has increased in this case due to the increase in all its factors. The increase in the adjusted risks of these states (Figure 4a) confirms this conclusion. These maps can be used for prioritizing national or regional adaptation planning for drought. The framework presented here can be used for future studies focusing on specific regions or outcomes of drought.

We have also included total risk and risk over individual vulnerability variables for each study period (Figure 4 and Figure 5). The consistent adjusted risks in Texas are higher than the national average, which indicates that this state needs special considerations during drought (Figure 4). Over the two time periods, there were considerable shifts of high hazard areas from the Central states to Western states. These results show the importance of evaluating the appropriate timespans of climatic factors for estimating risk. Within our study, a single decadal approach would not adequately capture this change. The West and Midwest produced the greatest change in total risk and the Northeast contains small areas of increased risk. Finally, our adjusted risk map can be useful for national-level discussion and planning, while information added in the supplementary section (and the provided dataset) provides a detailed map of the counties that are in the high risk (High|High) condition in each case.

The limitations of this study come from the complex nature of drought and the potential outcomes. Health vulnerability to drought can be regionally specific. While most drought risk studies focus on agricultural or water shortages, the complicated paths of health effects make choosing appropriate indicators more challenging [9]. For example, several studies on health relationships with drought found different effects based on gender [23,55]. However, we did not consider gender stratification in our analysis because of a recent study in the United States that did not find these differences [56]. The same limitation also occurs with the selection of the hazard indicator. We used USDM due to its widespread use for decision-making on drought and its categorical formatting that better fits our qualitative model. However, there are many other drought indicators, and identifying drought characteristics that are associated with health outcomes at different exposure levels with drought indicators remains unexplored [55,57,58]. Different areas marked as high risk in this study might have different adaptive capacities in place (such as water management strategies for drought condition or the level of medical care) that will make the actual effects of drought much lower than areas with lower risk but without existing mechanisms. Applying those measures in this national-level study is difficult and was not aligned with the purpose of this study. For studies at the regional or local level, we suggest including adaptive capacities and resilience [59]. Due to the categorical classification, and due to normalizing the hazards, the distinctions among high-risk areas may be captured through numerical models. However, the focus of this study was at the national level to look for generalizable patterns and provide opportunities for more detailed studies in smaller regions of concern. It is also worth noting that the Empirical Bayesian (EB) standardization is suggested for spatial autocorrelation analyses of ratio variables [60,61]. This is considered a remedy for the variance instability of such variables. The resulting maps may show more differences from the standard method in areas where variations among values for the base variables (e.g., the population at risk) are higher [45]. Due to the mixing of the variables in this study, we were not able to apply this correction.

Addressing the limitations as identified will provide immediate improvements for future regional studies. More quantitative analysis of health outcomes of drought will also improve future vulnerability mapping. Our results will hopefully provide opportunities to identify areas of higher concern to concentrate efforts for future health studies. In addition, risk models can reveal higher details for estimating the health costs of droughts for decision making. Extending the risk assessment into different future scenarios with uncertainty quantification is a critical task.

## 5. Conclusions

As drought manifests differently than many other climate-related disasters, the resulting health impact can cascade from issues associated with food insecurity, water availability, infrastructural issues, environmental degradation, and economic effects [23]. Although all populations have some associated risk from drought, certain populations are more at-risk than others. Evaluating and measuring the health-associated vulnerabilities of drought provide opportunities to identify spatial and temporal patterns in associated risk. Our results indicate that the risks and hazards associated with drought are in constant flux and must be reevaluated periodically. These results provide a guide for future focused studies on risks and their components. This information on the importance of reevaluating drought vulnerability with climate change is useful for public health professionals and others interested in health outcomes associated with climate-related disasters.

## Figures and Tables

**Table 1:** Significant changes in percentage of the vulnerable population from 2010~2014 to 2015~2019. Positive values show an increase from the first 5-year period to the second study period, and negative values indicate a decrease in percentage.

State	Over 65	Poverty	Under 5
Alabama	2.56	−1.94	−0.14
Arkansas	1.84	−1.49	-
Colorado	2.58	-	-
Florida	2.47	−2.07	−0.29
Georgia	2.05	-	−0.38
Illinois	1.62	-	-
Indiana	2.03	-	-
Iowa	1.42	-	-
Kentucky	2.08	-	-
Louisiana	2.01	-	-
Maryland	1.96	-	-
Michigan	2.47	−1.39	-
Minnesota	-	−1.25	-
Mississippi	1.96	-	−0.49
Missouri	1.71	−1.80	-
Nebraska	-	−1.00	-
New Jersey	3.13	-	-
New York	2.29	-	-
North Carolina	2.81	−2.36	−0.40
Ohio	1.87	−1.36	-
Oklahoma	1.30	-	-
Oregon	-	−3.20	-
Pennsylvania	1.92	-	-
South Caroli-	2.63	-	−0.46
Tennessee	2.18	−1.81	-
Texas	1.41	−1.32	-
Utah	-	-	−0.84
Virginia	2.21	-	-
West Virginia	2.75	-	-
Wisconsin	2.13	−1.12	-

## Data Availability

The data for socioeconomic vulnerability variables can be found through API of American Community Survey [62]. Data for community access to safe drinking water are openly available at Safe Drinking Water Information System (SDWIS) [63]. Land cover raster maps of 2019 and 2016 can be found at Multi-Resolution Land Characteristic Consortium (MRLC) website [64]. To access drought data, we used the Comprehensive Statistics page of Data Download section of U.S. Drought Monitor [65]. The data that support the findings of this study are openly available in Mendeley Data [66].

## Supplementary Materials

The following supporting information can be downloaded at: https://www.mdpi.com/article/10.3390/ijerph19084628/s1, Figure S1: Flowcharts of the processes in the methods section, Figure S2: Visualization of Table 1. Grey areas have no significant changes between the two study periods, Figure S3: Boxplot maps of changes in vulnerability ratios (Second period–First Period). The hinges for the boxplot are considered as 1.5 times of interquartile range below minimum and above maximum values, Figure S4: Map of two hazard parameters for each study period. Left contains the first five-year period and left side shows their equivalents for the second study period, Figure S5: Counties with High|High values in bivariate Local Moran’s I analysis. Vulnerability is the central variable, and the hazard is used as spatial lag in Local Moran’s formulation. First two rows are for interaction with hazard duration indicator, and two last rows are interactions with hazard frequency indicator, Table S1: Approximated total number of counties in each class of clustering for each vulnerability, Table S2: Relative risk for each state and vulnerability measure, corresponding to Figure 4. The baseline is national ratio of counties in the corresponding vulnerability that are in High|High category of bivariate LISA analysis. Columns with I represent first study period, and columns with II are for the second study period.
