# Supplementary material for: Evaluating Changes in Health Risk from Drought over the Contiguous United States
Source: Int J Environ Res Public Health. 2022 Apr 12;19(8):4628. doi: 10.3390/ijerph19084628 (PMC9031499; doi:10.3390/ijerph19084628)
Supplement: Supplementary file 1 [file ijerph-19-04628-s001.zip › ijerph-1628007-supplementary.pdf]

Supplementary Material for *Evaluating Changes in Health Risk from Drought Over the Contiguous United States*

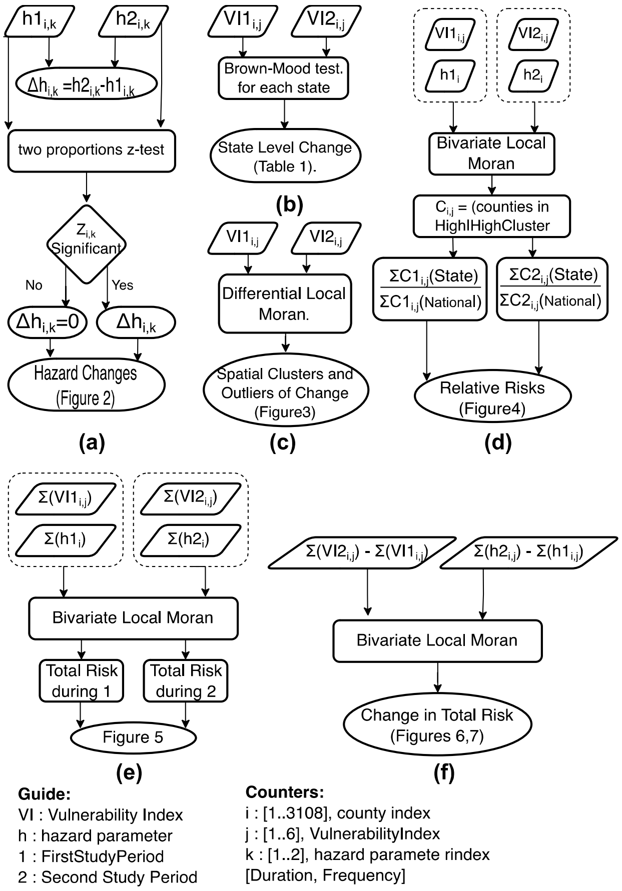

Figure S1. Flowcharts of the processes in the methods section. (**a**) Calculating hazard changes, (**b**) Calculating state level changes in VIs, (**c**) Calculating Spatial cluster and outliers of VI changes over the study periods, (**d**) Calculating adjusted risk levels for each VI and study period, (**e**) Calculating total risk in each study period, (**f**) Calculating change in the total risk over the study periods.

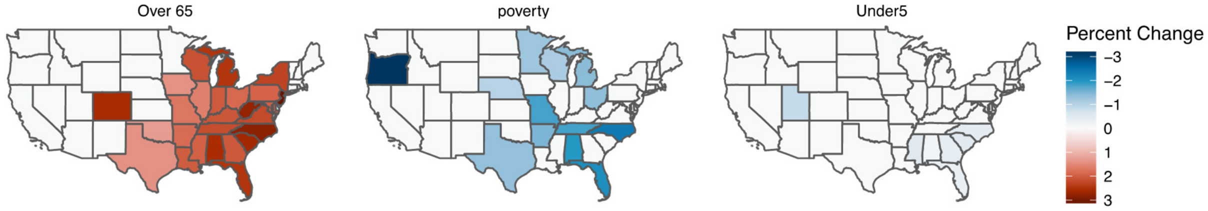

Figure S2. Visualization of Table 1. Grey areas have no significant changes between the two study periods.

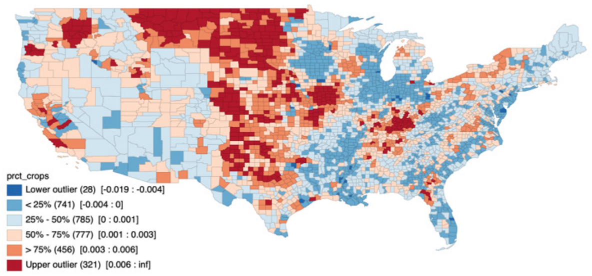

(a) Crops

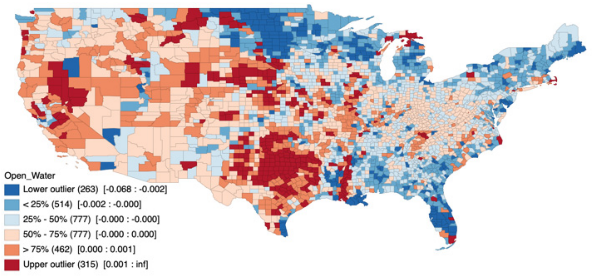

(b) Open Water

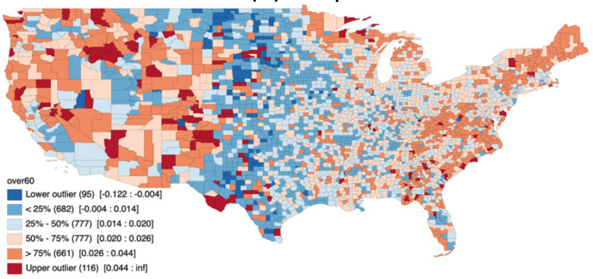

(c) Over 65

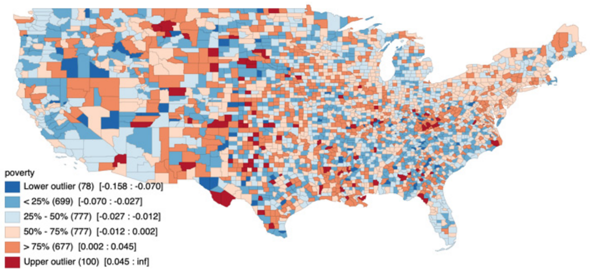

(d) Poverty

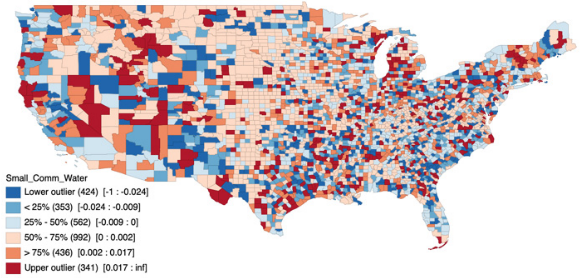

(e) Small Community Water

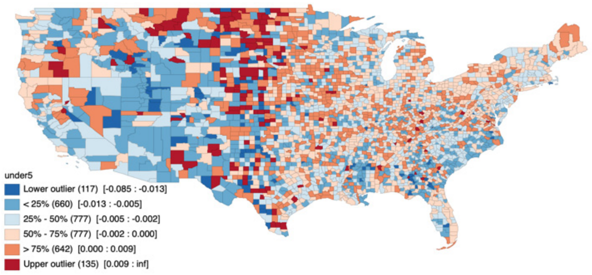

(f) Under 5

Figure S3. Boxplot maps of changes in vulnerability ratios (Second period – First Period). The hinges for the boxplot are considered as 1.5 times of interquartile range below minimum and above maximum values. (**a**) ratio of croplands, (**b**) ratio of open water lands, (**c**) ratio of Over 65-year-old population, (**d**) ratio of population below poverty line, (**e**) ratio of the small communities depending on self-supplied water, (**f**) ratio of Under 5-year-old population.

Table S1. Approximated total number of counties in each class of clustering for each vulnerability.

Type	Crops	Open Water	Over 65	Poverty	Small Community Water	Under 5
Group Increase	217	110	38	46	18	33
Group Decrease	133	68	61	34	12	34
Single Decrease	8	*0*	13	28	10	18
Single Increase State	5	8	19	17	32	21

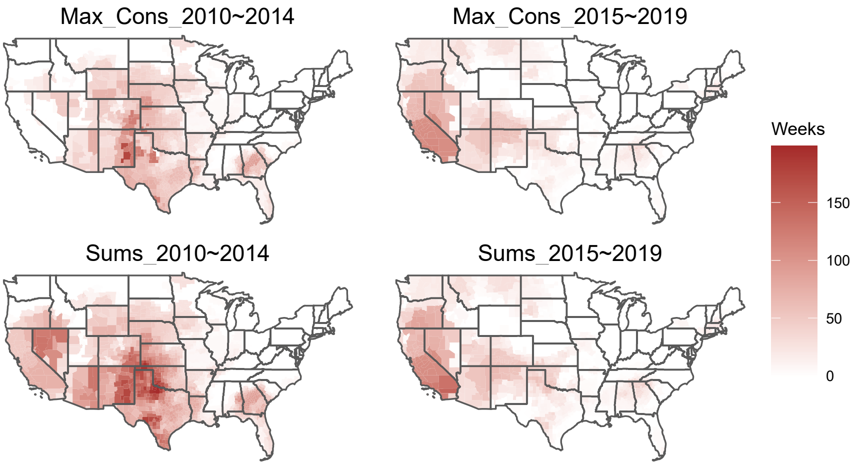

Figure S4. Map of two hazard parameters for each study period. Left contains the first five-year period and left side shows their equivalents for the second study period.

Table S2. Relative risk for each state and vulnerability measure, corresponding to Figure 3. The baseline is national ratio of counties in the corresponding vulnerability that are in High-High category of bivariate LISA analysis. Columns with I represent first study period, and columns with II are for the second study period.

	Crops		Open Water		Over 65		Poverty		Small Community Water		Under 5	
	I	II	I	II	I	II	I	II	I	II	I	II
Alabama	0.52	0.74	2.07	6.17	0.77	3.01	1.69	5.38	0.16	0.86	0.68	3.33
Arizona	0.29	0.82	-	-	2.2	4.28	3.13	5.25	1.95	2.95	3.26	3.93
Arkansas	0.23	0.16	0.79	0.37	0.5	0.31	0.83	0.55	0.39	0.35	0.47	0.39
California	1.06	4.05	2.5	5.13	1.14	3.32	1.62	3.72	0.82	2.44	1.32	4.35
Colorado	1.85	1.26	-	0.52	2.21	3.08	2.14	2.27	2.62	3.53	1.75	1.12
Connecticut	-	-	-	4.82	-	1.15	-	-	-	1.11	-	-
Florida	0.13	-	0.49	-	0.21	-	0.47	-	0.22	-	0.26	-
Georgia	3.06	0.51	4.81	3.08	1.51	1.7	4.4	3.1	2.57	1.31	3.01	2.17
Idaho	-	0.98	-	0.5	-	1.88	0.09	1.13	0.08	1.61	0.08	1.05
Iowa	0.22	-	-	-	0.14	-	0.04	-	0.11	-	0.11	-
Kansas	5.31	3.53	1.32	1	4.49	1.48	0.86	0.59	3.51	1.6	2.46	1.76
Louisiana	0.82	0.19	3.71	1.03	0.88	0.5	2.63	0.84	1.6	0.48	2.02	0.53
Massachusetts	-	-	-	4.72	-	-	-	0.59	-	-	-	-
Minnesota	0.25	-	0.15	-	0.16	-	0.04	-	0.13	-	0.08	-
Mississippi	-	0.75	-	0.87	-	0.22	-	0.81	-	0.54	-	0.97
Missouri	-	1.61	-	0.62	-	0.76	-	0.5	-	0.81	-	0.88
Monta-	-	3.64	-	1.57	0.08	4.17	-	1.7	0.07	4.51	-	2.48

Nebraska	4.63	-	1.92	-	4.77	-	0.46	-	3.65	-	2.37	-
Nevada	-	-	1.94	3.24	2.22	4.85	1.38	1.46	2.15	4.69	1.64	2.47
New Hampshire	-	-	-	2.2	-	-	-	-	-	0.89	-	-
New Mexico	0.53	0.75	-	-	4.29	4.17	6.16	4.52	3.76	4.02	2.96	2.55
New York	-	0.5	-	0.71	-	0.37	-	0.07	-	0.57	-	-
North Caroli-	-	-	-	0.44	-	0.69	-	0.46	-	0.58	-	0.08
North Dakota	-	4.08	-	2.18	-	1.3	-	0.31	-	2.17	-	2.14
Oklahoma	3.71	4.49	3.69	2.15	3.55	2.38	2.54	2.8	3.03	2.87	4.04	2.95
Oregon	-	1.89	-	1.68	0.13	4.33	0.22	2.65	0.2	2.83	0.1	2.34
South Caroli-	0.48	-	1.72	1.2	0.62	0.4	1.53	0.81	0.56	-	0.61	0.46
South Dakota	1.46	0.75	1.3	0.33	1.21	0.49	0.83	0.31	1.55	0.8	1.16	0.7
Tennessee	-	0.26	-	1.22	-	1.11	-	1.13	-	0.09	-	0.27
Texas	3.35	3.26	3.59	1.63	3.55	2.06	4.06	2.14	3.81	2.74	4.11	2.48
Utah	-	-	0.23	1.9	0.16	1.58	0.4	1.71	0.5	3.66	0.6	5.51
Washington	-	4.44	-	3.82	-	2.23	-	1.81	-	2.84	-	2.37
Wyoming	0.76	-	-	-	2.05	0.4	0.51	-	2.22	0.38	2.58	0.37

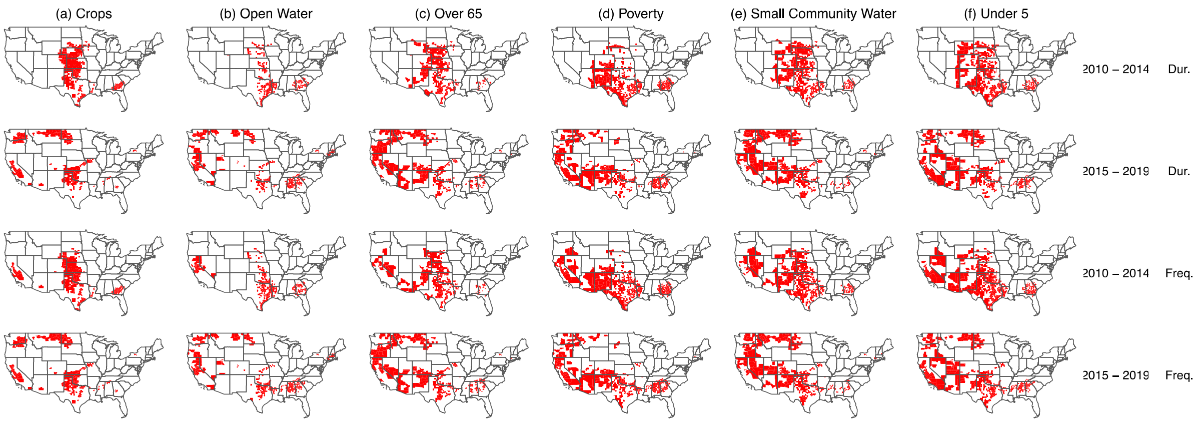

Figure S5. Counties with High-High values in bivariate local Moran's I analysis. Vulnerability is the central variable, and the hazard is used as spatial lag in local Moran's formulation. First two rows are for interaction with Hazard Duration indicator, and two last rows are interactions with Hazard Frequency Indicator. (**a**) ratio of croplands, (**b**) ratio of open water lands, (**c**) ratio of Over 65-year-old population, (**d**) ratio of population below poverty line, (**e**) ratio of the small communities depending on self-supplied water, (**f**) ratio of Under 5-year-old population.
